# A lanthanum coordination polymer with 3,6-di­chloro­phthalate and 2,4-di­chloro-6-(eth­oxy­carbon­yl)benzoate as ligands

**DOI:** 10.1107/S2056989025009508

**Published:** 2025-11-06

**Authors:** Christine Hénaff, Thierry Roisnel, Chloé Blais, Olivier Guillou, Carole Daiguebonne

**Affiliations:** a Univ Rennes, INSA Rennes, CNRS UMR 6226 "Institut des Sciences Chimiques de Rennes", 35708 Rennes, France; b Univ Rennes, CNRS UMR 6226 "Institut des Sciences Chimiques de Rennes" 35042 Rennes France; c Institut Universitaire de France, 1 rue Descartes 75005 Paris France; Universidade Federal do ABC, Brazil

**Keywords:** crystal structure, lanthanum, 3,6-di­chloro­phthalate, coordination polymer, luminescent marker

## Abstract

A one-dimensional lanthanum-based coordination polymer based on 3,6-di­chloro­phthalate has been prepared and structurally described, namely, poly[[tetra­aqua­[2,4-di­chloro-6-(eth­oxy­carbon­yl)benzoato](μ_3_-3,6-di­chloro­phthal­ato)lanthanum(III)] monohydrate], {[La(C_8_H_2_Cl_2_O_4_)(C_10_H_7_Cl_2_O_4_)(H_2_O)_4_]·H_2_O}_∞_. Its crystal structure can be described based on mol­ecular double chains in which lanthanum ions are linked to each other by ligands. There are several structural features that seem promising as far as luminescence properties are concerned, such as the presence of strong hydrogen bonds and of short halogen contacts, as well as quite long inter­metallic distances. It is a pity that, to date, only a lanthanum-based compound has been obtained.

## Abstract 2

A one-dimensional lanthanum coordination polymer based on 3,6-di­chloro­phthalate has been prepared by microwaves-assisted synthesis and structurally described.

## 1. Chemical context

Materials traceability is an ongoing challenge. Indeed, the consumption of plastics is continuously growing worldwide, and their recycling is an environmental emergency. However, whatever the recycling process, homogeneous waste batches are required and marking plastics would enable rigorous waste sorting (Vollmer *et al.*, 2020 ▸). However, plastics must not only be marked according to the polymer matrix but also according to their complete formulation. Therefore, because of the wide variety of plastic formulations, marking them requires a large number of markers.

Our group was the first to design luminescent hetero-lanthanide coordination polymers (Kerbellec *et al.*, 2009 ▸) and to demonstrate that these compounds behave like true mol­ecular alloys (Blais *et al.*, 2023 ▸; Ferlay & Hosseini, 2004 ▸; Haquin *et al.*, 2013 ▸). Luminescent markers based on lanthanide coordination polymers have proved their efficiency in the fight against counterfeiting (Guillou *et al.*, 2016 ▸). They could also be relevant for marking plastics to improve their recyclability (Daiguebonne *et al.*, 2025 ▸).

**Figure 1:**
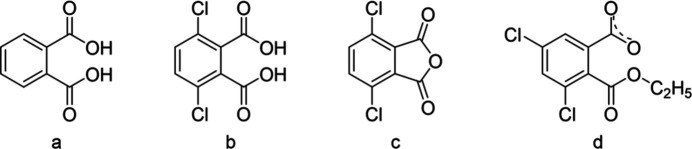
Schematic representations of benzene-1,2-di­carb­oxy­lic or phthalic acid (*a*), 3,6-di­chloro­phthalic acid (*b*), 4,7-di­chloro­benzo­furan-1,3-dione (*c*) and 2,4-di­chloro-6-(eth­oxy­carbon­yl)benzoate (*d*).

**Figure 2:**
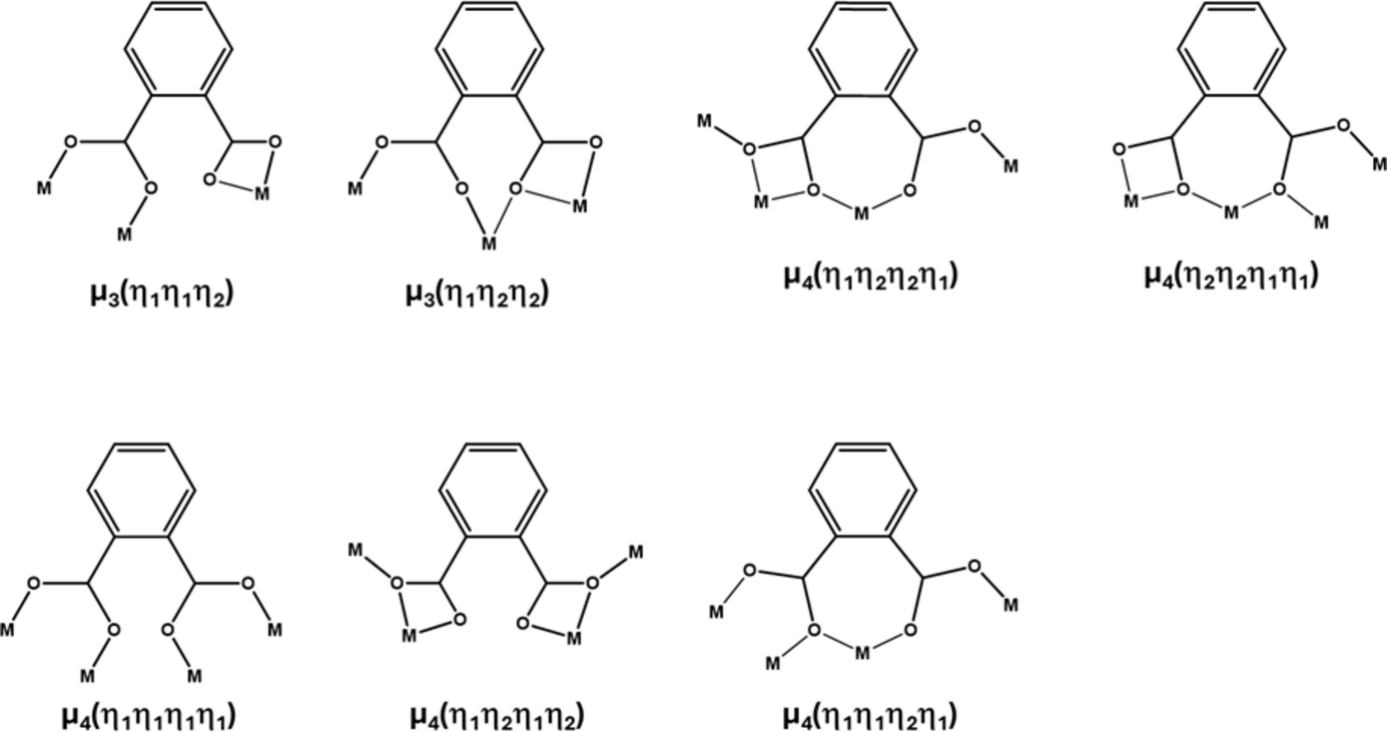
Coordination modes observed in lanthanide coordination polymers based on a phthalate ligand.

Recent studies strongly suggest that hetero-lanthanide coordination polymers with halogeno-derivatives of phthalic acid (Fig. 1 ▸) exhibit very promising luminescent properties and could be suitable for materials traceability (Blais *et al.*, 2025 ▸; Ngom *et al.*, 2024 ▸; Pointel, Suffren *et al.*, 2020 ▸; Pointel, Houard *et al.*, 2020 ▸; Hénaff *et al.*, 2026 ▸). There are several reasons that can explain such promising luminescent properties (Bünzli, 2010 ▸, 2015 ▸): (i) the adjacent positions of the two carboxyl­ate functions enable the ligand to bridge several metal ions (Fig. 2 ▸), which can induce a fairly high rigidity of the mol­ecular motif and therefore helps to limit non-radiative vibrational de-excitation; (ii) halogeno substituents can be involved in halogen-bond networks (Cavallo *et al.*, 2016 ▸; Fourmigué, 2009 ▸) that can keep mol­ecular motifs away from each other and prevent π-stacking inter­actions, which is beneficial for reducing inter­metallic energy transfers (Förster, 1960 ▸; Dexter, 1953 ▸; Blais *et al.*, 2022 ▸; Imbert *et al.*, 2003 ▸). The nature and the position of the halogeno substituents influence the energy of the first singlet and triplet excited states (Latva *et al.*, 1997 ▸; Steemers *et al.*, 1995 ▸), the photo-induced electron transfer (PET) mechanism (Freslon *et al.*, 2014 ▸) and the strength of the halogen inter­actions (Metrangolo *et al.*, 2008 ▸; Metrangolo & Resnati, 2001 ▸). However, contrary to halogenoterephthalate-based lanthanide coordination polymers (Smith *et al.*, 2024 ▸), halogenophthalate-based lanthanide coordination polymers have been little studied. So, for example, the only lanthanide coordination polymers based on di­chloro­phthalates described today are those involving 4,5-di­chloro­phthalate (Badiane *et al.*, 2018 ▸; Qiao *et al.*, 2018 ▸; He *et al.*, 2017 ▸) and, to the best of our knowledge, there is no example of lanthanide coordination polymers based on 3,6-di­chloro­phthalate (3,6-dcpa^2−^) in the literature.

We have thus undertaken a study of such compounds based on 3,6-di­chloro­phthalate. For reasons of commercial availability, and because anhydrides easily hydrolyse leading to the corresponding carb­oxy­lic acids, we have chosen to use 4,7-di­chloro­benzo­furan-1,3-dione as starting reactant. In the frame of this study, we have obtained a lanthanum coordination polymer with chemical formula [La(3,6-dcpa)(C_10_H_7_Cl_2_O_4_)(H_2_O)_4_·H_2_O]_∞_. In this compound, as expected, 3,6-di­chloro-phthalate comes from 4,7-di­chloro­benzo­furan-1,3-dione. Unexpectedly, a second ligand is also produced: 2,4-di­chloro-6-(eth­oxy­carbon­yl)benzoate. This kind of re-organization of the ligand is commonly observed (Feng *et al.*, 2016 ▸; Abdallah *et al.*, 2020 ▸). To the best of our knowledge, this compound constitutes the first example of a lanthanide coordination polymer based on the 3,6-dcpa^2−^ ligand. Despite great synthetic effort, to date we have not not succeeded in synthesizing isomorphous compounds that involve luminescent lanthanide ions.
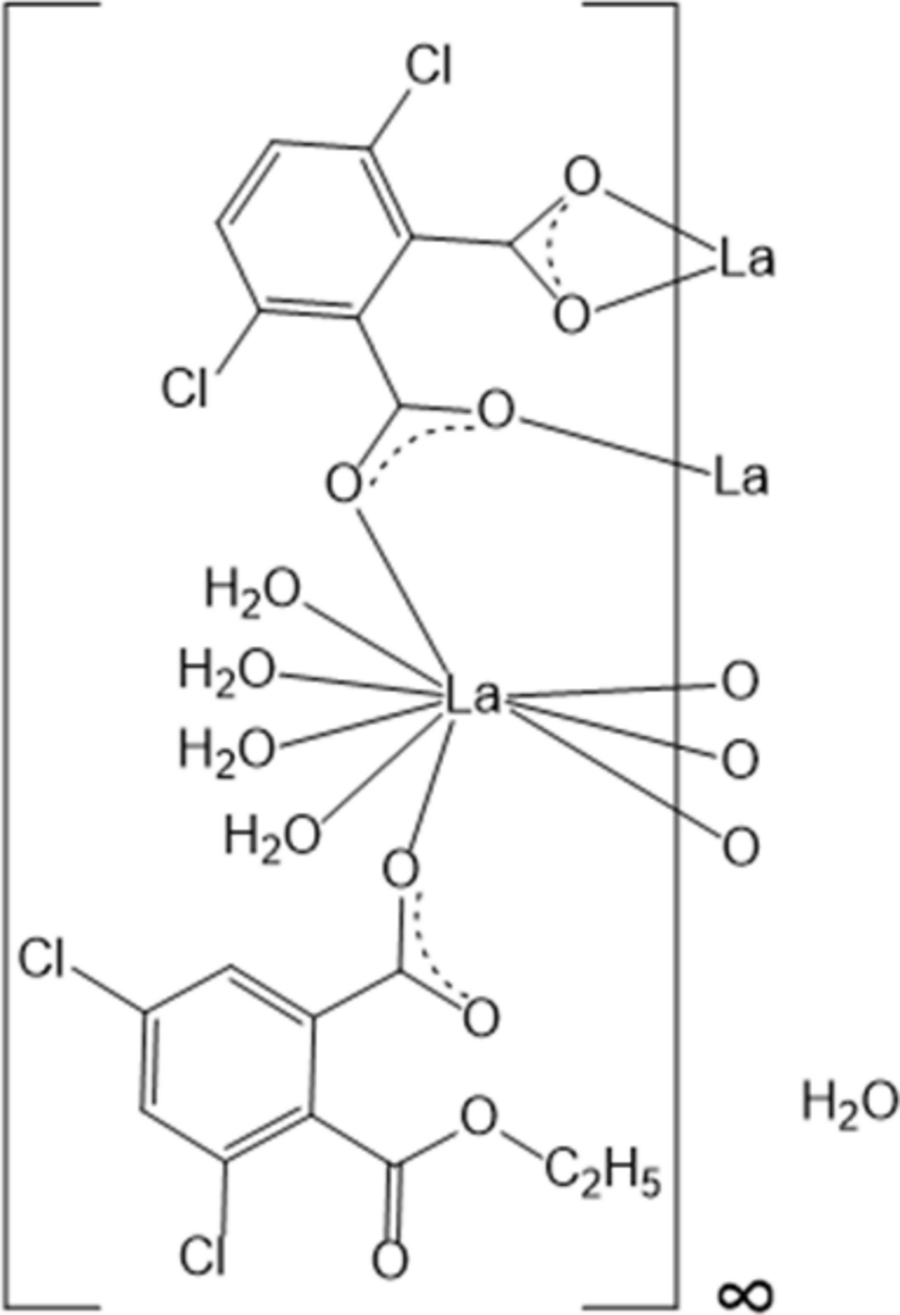


## 2. Structural commentary

**Figure 3:**
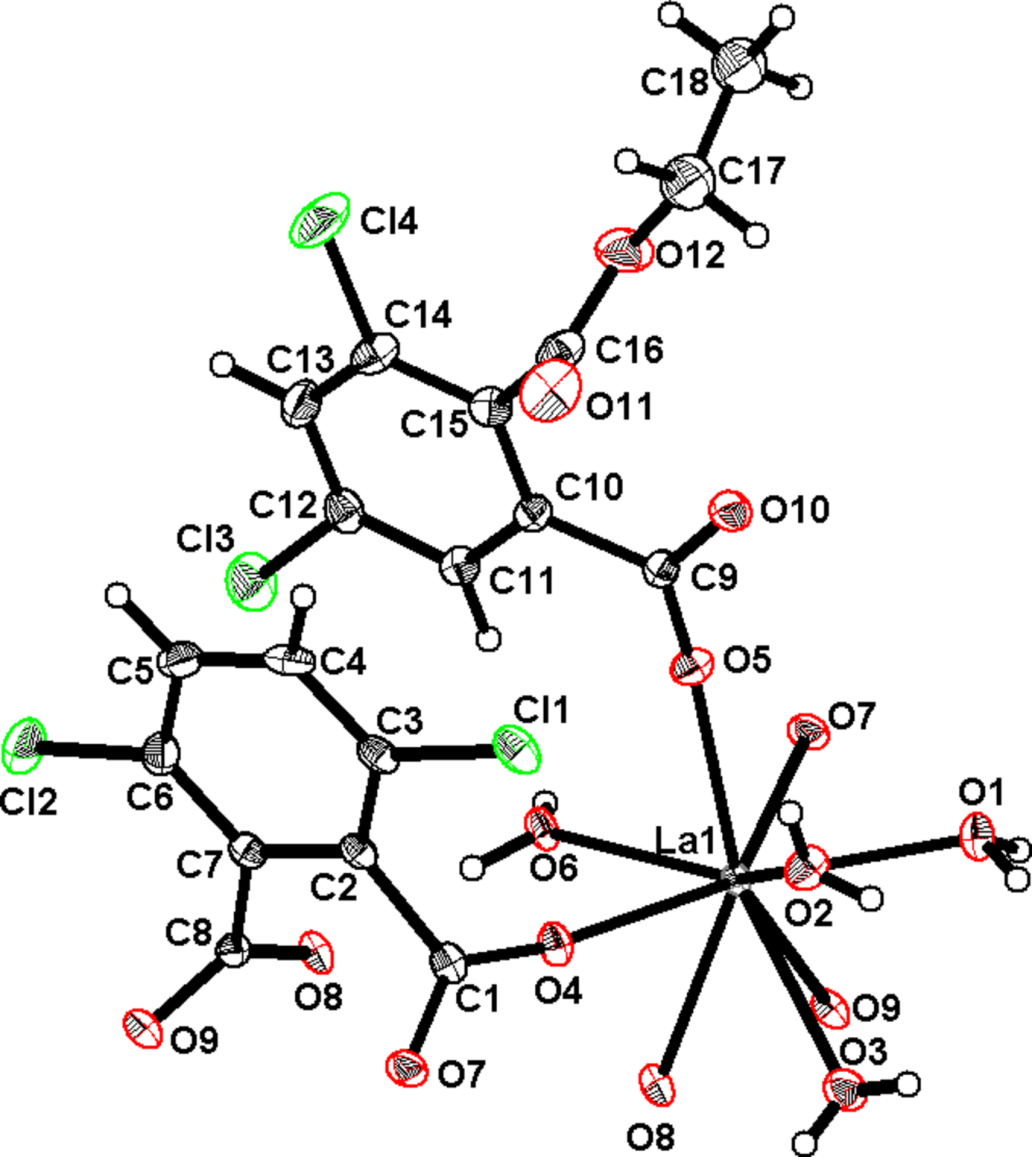
Projection view of an extended asymmetric unit with the numbering scheme of [La(3,6-dcpa)(C_10_H_7_Cl_2_O_4_)(H_2_O)_4_·H_2_O]_∞_. Displacement ellipsoids are drawn at the 50% probability level. [Symmetry codes: (i) −1 + *x*, *y*, *z*; (ii) 1 − *x*, 1 − *y*, 2 − *z*]. The crystallization water mol­ecule is omitted.

Microwave-assisted reaction in water between lanthanum chloride and 4,7-di­chloro­benzo­furan-1,3-dione leads to a coordination polymer with chemical formula [La(3,6-dcpa)(C_10_H_7_Cl_2_O_4_)(H_2_O)_4_·H_2_O]_∞_ where 3,6-dcpa^2−^ symbolizes 3,6-di­chloro­phtalate (CCDC-2430840) (Fig. 3 ▸).

**Figure 4:**
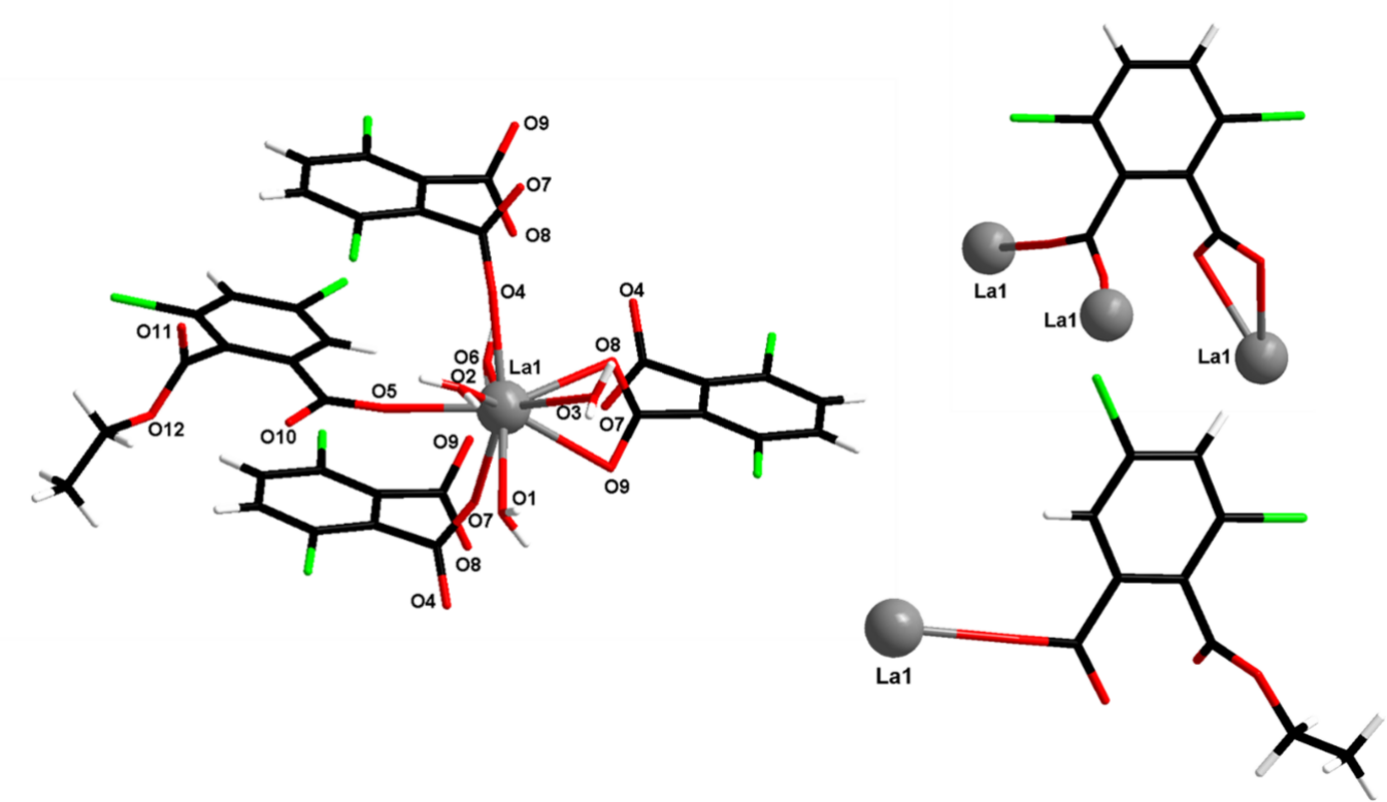
Schematic representation of the neighbourhood of the La^3+^ ion (left) and coordination modes of the 3,6-dcpa^2−^ ligand (top right) and of the 2,4-di­chloro-6-(eth­oxy­carbon­yl)benzoate ligand (bottom right) of [La(3,6-dcpa)(C_10_H_7_Cl_2_O_4_)(H_2_O)_4_·H_2_O]_∞_.

There is one independent lanthanum ion in this crystal structure. It is ninefold coordinated by nine oxygen atoms. Four out of the nine are from four coordination water mol­ecules (O1, O2, O3 and O6), four more (O4, O7^i^, O8^ii^ and O9^ii^) are from carboxyl­ate functions that belong to three different 3,6-dcpa^2−^ ligands and the remaining one (O5) is from a carboxyl­ate function that belongs to the 2,4-di­chloro-6-(eth­oxy­carbon­yl)benzoate ligand. They form a spherical capped square anti­prism (Table S1) (Casanova *et al.*, 2005 ▸; Alvarez *et al.*, 2005 ▸). There is also one independent 3,6-dcpa^2−^ ligand and one independent 2,4-di­chloro-6-(eth­oxy­carbon­yl)benzoate ligand in the crystal structure. The former is μ_3_(η_1_η_1_η_2_) and the latter is μ_1_(η_1_) (Fig. 4 ▸) Finally, there is one crystallization water mol­ecule (H13*A*–O13–H13*B*) in the crystal structure.

**Figure 5:**
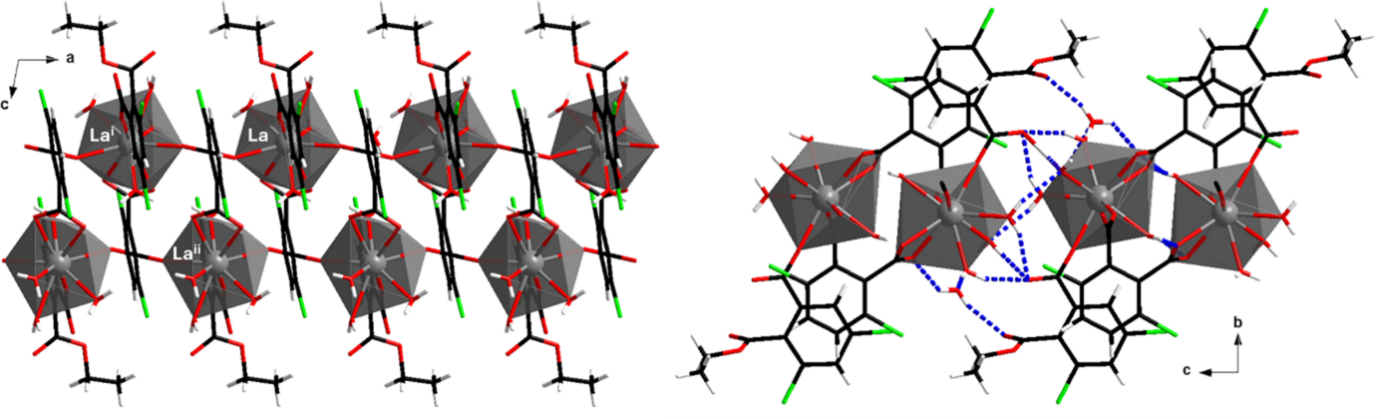
Left: Projection view along the *b* axis of a mol­ecular double chain of [La(3,6-dcpa)(C_10_H_7_Cl_2_O_4_)(H_2_O)_4_·H_2_O]_∞_. Shortest inter­metallic distances are *d*(La—La^i^) = 6.8045 (8) Å, *d*(La—La^ii^) = 6.0680 (4) Å and *d*(La^i^—La^ii^) = 6.3651 (3) Å [Symmetry codes: (i) −1 + *x*, *y*, *z*; (ii) −*x*, 1 − *y*, 2 − *z*]. Right: Projection view along the *a* axis of two adjacent mol­ecular motifs. Dotted blue lines indicate hydrogen bonds.

The crystal structure is mono-dimensional and can be described based on mol­ecular double chains that spread parallel to the *a* axis (Fig. 5 ▸). Inside these mol­ecular double chains, lanthanum ions are linked to each other by 3,6-dcpa^2−^ ligands while the ethyl groups of the 2,4-di­chloro-6-(eth­oxy­carbon­yl)benzoates point toward the inter­molecular space. The shortest distances between lanthanide ions that belong to the same mol­ecular motif are about 6 Å (Fig. 5 ▸).

It is noticeable that there is a dense network of strong intra- and inter-mol­ecular hydrogen bonds (Fig. 5 ▸ and Table 1 ▸) in this crystal structure. Additionally, there are some weak π–π inter­actions (shortest centroid–centroid distances are 3.7 Å).

## 3. Supra­molecular features

**Figure 6:**
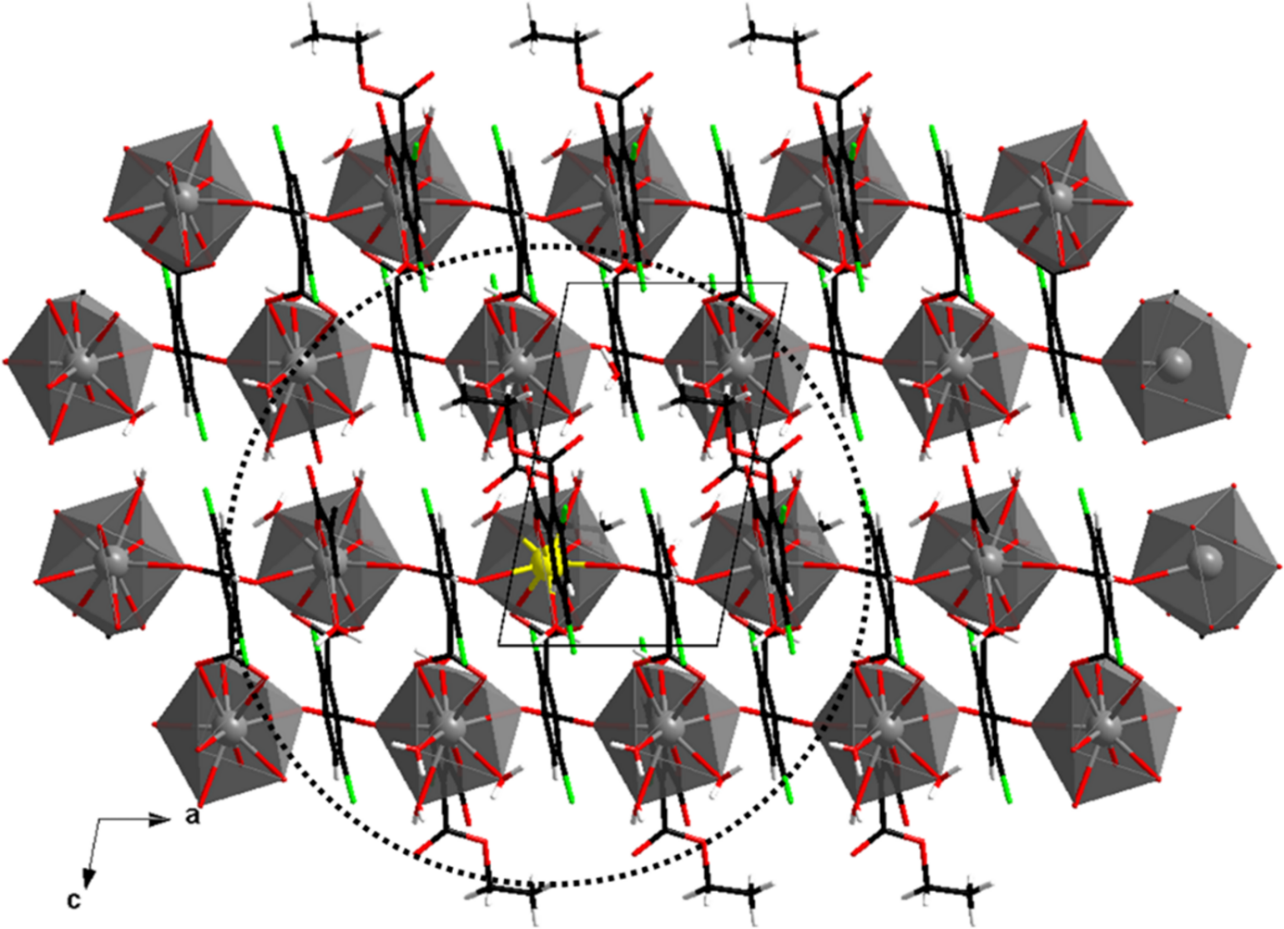
Projection view along the *b* axis of two adjacent mol­ecular motifs. The dotted circle centred on the given La^3+^ ion (in yellow) has a 10 Å radius.

The crystal structure can be described as a juxtaposition of mol­ecular chains that spread along the *a*-axis direction. Beyond the network of strong hydrogen bonds, the cohesion of the crystal packing is reinforced by Cl⋯Cl inter­actions (Table 2 ▸). There are seven La^3+^ ions closer than 10 Å from a given La^3+^ ion (Table 3 ▸). All seven belong to two adjacent mol­ecular motifs spreading parallel to the *ac* plane (Fig. 6 ▸).

In conclusion, this crystal structure presents inter­esting features as far as luminescent properties are concerned. Indeed, the lanthanide ions are quite far from each other and the dense network of hydrogen and halogen bonds is expected to limit non-radiative vibrational de-excitation. The reproducibility of the synthesis was checked by reproducing it several times. Unfortunately, to date, we have not succeeded in synthesizing any iso-structural coordination polymer based on a luminescent lanthanide ion.

## 4. Database survey

A search of the Cambridge Structural Database was performed using ConQuest (version 2024.2.0, CSD version 5.45, updated September 2024; Groom *et al.*, 2016 ▸). For lanthanide coordination polymers based on phthalate ligands, see: Li *et al.* (2009 ▸; CSD refcode KUGPUY); Meng *et al.* (2006 ▸; LEJPIA); Wan *et al.* (2002 ▸; WUJSID, WUJSOJ, WUJSUP, WUJTAW); Song *et al.* (2004 ▸, 2010 ▸; FIBWOD, YURGIC); Thirumurugan & Natarajan (2003 ▸; BEVPIC); Wang *et al.* (2008 ▸; FARJAL, KIWPOW) Pizon *et al.* (2010 ▸; IJEJOX); Luo *et al.* (2010 ▸; DUWRIX; Lush & Shen (2011 ▸; AZITOT); for 3,6-di­chloro­phthalate, see: Mattes & Dorau (1986 ▸; SAZQOZ); for lanthanide coordination polymers based on 4,5-di­chloro-hthalates, see: Badiane *et al.* (2018 ▸; BETYOR); Qiao *et al.* (2018 ▸; GIQCIV, GIQCOB, GIQCUH, GIQDAO, MICCEJ); He *et al.* (2017 ▸; DEJGAD). For a structural comparison between these crystal structures, see: Hénaff *et al.* (2026 ▸).

## 5. Synthesis and crystallization

Lanthanum oxide (4N) was purchased from Ampère. Hydrated lanthanum chloride [LaCl_3_(H_2_O)_6_] was prepared according to established procedures (Desreux, 1989 ▸). 4,7-Di­chloro­benzo­furan-1,3-dione (C_8_H_2_Cl_2_O_3_, 98%) was purchased from BDLpharm and used without further purification. 0.5 mmol (185.6 mg) of LaCl_3_(H_2_O)_6_, 0.75 mmol (162.7 mg) of C_8_H_2_Cl_2_O_3_, 1.5 mL of a solution of sodium hydroxide (1 mol L^−1^) and 3.5 mL of deionized water were put in a 10 mL sealed Pyrex test tube in a CEM Discover microwave oven and maintained for 10 min under stirring (*T* = 403 K; *P* = 2.5 bar). Single crystals suitable for X-ray diffraction were obtained after slow evaporation of the supernatant solution extracted after the synthesis.

## 6. Refinement

Crystal data, data collection and structure refinement details are summarized in Table 4 ▸. Except for O-bound H atoms that were introduced in the structural model through Fourier difference map analysis, H atoms were finally included in their calculated positions (C—H = 0.95–0.98 Å) and treated as riding on their parent atom with *U*_iso_(H) = 1.2–1.5*U*_eq_(C).

## Supplementary Material

Crystal structure: contains datablock(s) I. DOI: 10.1107/S2056989025009508/ee2020sup1.cif

Structure factors: contains datablock(s) I. DOI: 10.1107/S2056989025009508/ee2020Isup3.hkl

CCDC reference: 2430840

Additional supporting information:  crystallographic information; 3D view; checkCIF report

## Figures and Tables

**Table 1:** Hydrogen-bond geometry (Å, °)

*D*—H⋯*A*	*D*—H	H⋯*A*	*D*⋯*A*	*D*—H⋯*A*
O1—H1*A*⋯O13	0.84 (4)	2.00 (4)	2.787 (4)	155 (4)
O1—H1*B*⋯O10^iii^	0.80 (4)	1.89 (5)	2.663 (4)	164 (4)
O2—H2*A*⋯O10^iii^	0.77 (5)	2.28 (5)	2.972 (4)	150 (4)
O2—H2*B*⋯O1^iii^	0.80 (5)	2.11 (5)	2.868 (4)	159 (4)
O3—H3*A*⋯O10^iii^	0.82 (5)	2.10 (5)	2.850 (4)	152 (4)
O3—H3*B*⋯O13^i^	0.76 (5)	2.13 (5)	2.883 (4)	174 (5)
O6—H6*A*⋯O9^iv^	0.78 (4)	2.10 (4)	2.864 (3)	166 (4)
O6—H6*B*⋯O8	0.85 (4)	1.92 (4)	2.772 (3)	175 (4)
O13—H13*A*⋯O11^iii^	0.81 (5)	2.16 (5)	2.948 (4)	164 (5)
O13—H13*B*⋯O9^v^	0.87 (5)	2.30 (5)	3.008 (4)	139 (4)

Symmetry codes: (i) 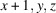; (iii) 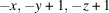; (iv) 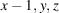; (v) 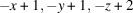.

**Table 2:** Selected interatomic distances (Å)

Cl2^i^⋯Cl3	3.4185 (16)	Cl3^ii^⋯Cl4	3.4645 (15)
Cl2⋯Cl3	3.4287 (15)		

Symmetry codes: (i) 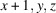; (ii) 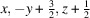.

**Table 3:** La⋯La distances (Å) shorter than 10 Å in [La(3,6-dcpa)(C_10_H_7_Cl_2_O_4_)(H_2_O)_4_·H_2_O]_∞_

Atom1	Atom2	Symmetry	Distance
La1	La1	−*x*, 1 − *y*, 2 − *z*	6.0682 (6)
	La1	−*x*, 1 − *y*, 1 − *z*	6.2554 (7)
	La1	1 − *x*, 1 − *y*, 2 − *z*	6.3650 (6)
	La1	1 + *x*, *y*, *z*	6.8044 (8)
	La1	−1 + *x*, *y*, *z*	6.8046 (8)
	La1	1 − *x*, 1 − *y*, 1 − *z*	8.5065 (8)
	La1	−1 − *x*, 1 − *y*, 1 − *z*	9.9248 (9)

**Table 4:** Experimental details

Crystal data	
Chemical formula	[La(C_8_H_2_Cl_2_O_4_)(C_10_H_7_Cl_2_O_4_)(H_2_O)_4_]·H_2_O
*M*_r_	724.04
Crystal system, space group	Monoclinic, *P*2_1_/*c*
Temperature (K)	150
*a*, *b*, *c* (Å)	6.8045 (7), 32.376 (3), 11.5188 (12)
β (°)	100.862 (4)
*V* (Å^3^)	2492.2 (4)
*Z*	4
Radiation type	Mo *K*α
μ (mm^−1^)	2.21
Crystal size (mm)	0.44 × 0.07 × 0.03

Data collection	
Diffractometer	D8 VENTURE Bruker AXS
Absorption correction	Multi-scan (*SADABS*; Krause *et al.*, 2015 ▸)
*T*_min_, *T*_max_	0.777, 0.936
No. of measured, independent and observed [*I* > 2σ(*I*)] reflections	19761, 5674, 5503
*R*_int_	0.028
(sin θ/λ)_max_ (Å^−1^)	0.650

Refinement	
*R*[*F*^2^ > 2σ(*F*^2^)], *wR*(*F*^2^), *S*	0.033, 0.068, 1.27
No. of reflections	5674
No. of parameters	355
H-atom treatment	H atoms treated by a mixture of independent and constrained refinement
Δρ_max_, Δρ_min_ (e Å^−3^)	0.72, −1.05

Computer programs: *APEX3* (Bruker, 2015 ▸), *SAINT* (Bruker, 2014 ▸), *SHELXT* (Sheldrick, 2015*a* ▸), *SHELXL2019/2* (Sheldrick, 2015*b* ▸), *SXGRAPH* (Farrugia, 1999 ▸), *Mercury* (Macrae *et al.*, 2020 ▸) and *CRYSCALC* (T. Roisnel, local program, 2024).

## supplementary crystallographic information

### Poly[[tetraaqua[2,4-dichloro-6-(ethoxycarbonyl)benzoato](µ3-3,6-dichlorophthalato)lanthanum(III)] monohydrate] Crystal data

**Table:**

[La(C_8_H_2_Cl_2_O_4_)(C_10_H_7_Cl_2_O_4_)(H_2_O)_4_]·H_2_O	*F*(000) = 1424
*M**_r_* = 724.04	*D*_x_ = 1.930 Mg m^−^^3^
Monoclinic, *P*2_1_/*c*	Mo *K*α radiation, λ = 0.71073 Å
*a* = 6.8045 (7) Å	Cell parameters from 9930 reflections
*b* = 32.376 (3) Å	θ = 2.6–27.5°
*c* = 11.5188 (12) Å	µ = 2.21 mm^−^^1^
β = 100.862 (4)°	*T* = 150 K
*V* = 2492.2 (4) Å^3^	Stick, colourless
*Z* = 4	0.44 × 0.07 × 0.03 mm

### Poly[[tetraaqua[2,4-dichloro-6-(ethoxycarbonyl)benzoato](µ3-3,6-dichlorophthalato)lanthanum(III)] monohydrate] Data collection

**Table:**

D8 VENTURE Bruker AXS diffractometer	5674 independent reflections
Radiation source: Incoatec microfocus sealed tube	5503 reflections with *I* > 2σ(*I*)
Multilayer monochromator	*R*_int_ = 0.028
Detector resolution: 7.39 pixels mm^-1^	θ_max_ = 27.5°, θ_min_ = 1.9°
rotation images scans	*h* = −8→8
Absorption correction: multi-scan (SADABS; Krause *et al.*, 2015)	*k* = −41→41
*T*_min_ = 0.777, *T*_max_ = 0.936	*l* = −14→14
19761 measured reflections	

### Poly[[tetraaqua[2,4-dichloro-6-(ethoxycarbonyl)benzoato](µ3-3,6-dichlorophthalato)lanthanum(III)] monohydrate] Refinement

**Table:**

Refinement on *F*^2^	Primary atom site location: dual
Least-squares matrix: full	Hydrogen site location: mixed
*R*[*F*^2^ > 2σ(*F*^2^)] = 0.033	H atoms treated by a mixture of independent and constrained refinement
*wR*(*F*^2^) = 0.068	*w* = 1/[σ^2^(*F*_o_^2^) + 7.126*P*] where *P* = (*F*_o_^2^ + 2*F*_c_^2^)/3
*S* = 1.27	(Δ/σ)_max_ = 0.002
5674 reflections	Δρ_max_ = 0.72 e Å^−^^3^
355 parameters	Δρ_min_ = −1.05 e Å^−^^3^
0 restraints	

### Poly[[tetraaqua[2,4-dichloro-6-(ethoxycarbonyl)benzoato](µ3-3,6-dichlorophthalato)lanthanum(III)] monohydrate] Special details

**Table:**

Experimental. A suitable crystal for X-ray diffraction single crystal experiment (colourless stick, dimensions = 0.030 x 0.070 x 0.440 mm) was selected and mounted with a cryoloop on the goniometer head of a D8 Venture (Bruker-AXS) diffractometer equipped with a CMOS-PHOTON70 detector, using Mo-K_α_ radiation (λ = 0.71073 Å, multilayer monochromator) at T = 150 (2) K. Crystal structure has been described in monoclinic symmetry and *P* 2_1_/*c* (I.T.#14) centric space group (R_int_=0.0280; R_sigma_=0.0262 ). Cell parameters have been refined as follows: *a* = 6.8045 (7) Å, *b* = 32.376 (3) Å, *c*=11.5188 (12) Å, *β* = 100.862 (4) °, *V* = 2492.2 (4) Å^3^. Number of formula unit Z is equal to 4 and calculated density d and absorption coefficient µ values are 1.930 g.cm^-3^ and 2.207mm^-1^ respectively. Crystal structure was solved by dual-space algorithm using *SHELXT* program and then refined with full-matrix least-squares methods based on F^2^ (*SHELXL* program). All non-Hydrogen atoms were refined with anisotropic atomic displacement parameters. Except Hydrogen atoms linked to Oxygen atoms that were introduced in the structural model through Fourier difference maps analysis, H atoms were finally included in their calculated positions and treated as riding on their parent atom with constrained thermal parameters. A final refinement on F^2^ with 5674 unique intensities and 355 parameters converged at ωR(F^2^)=0.0678 (R_F_ = 0.0325) for 5503 observed reflections with I > 2σ.
Geometry. All esds (except the esd in the dihedral angle between two l.s. planes) are estimated using the full covariance matrix. The cell esds are taken into account individually in the estimation of esds in distances, angles and torsion angles; correlations between esds in cell parameters are only used when they are defined by crystal symmetry. An approximate (isotropic) treatment of cell esds is used for estimating esds involving l.s. planes.
Refinement. Refinement of F^2^ against ALL reflections. The weighted R-factor wR and goodness of fit S are based on F^2^, conventional R-factors R are based on F, with F set to zero for negative F^2^. The threshold expression of F^2^ > 2sigma(F^2^) is used only for calculating R-factors(gt) etc. and is not relevant to the choice of reflections for refinement. R-factors based on F^2^ are statistically about twice as large as those based on F, and R- factors based on ALL data will be even larger.

### Poly[[tetraaqua[2,4-dichloro-6-(ethoxycarbonyl)benzoato](µ3-3,6-dichlorophthalato)lanthanum(III)] monohydrate] Fractional atomic coordinates and isotropic or equivalent isotropic displacement parameters (Å^2^)

**Table:**

	*x*	*y*	*z*	*U*_iso_*/*U*_eq_	
La1	0.15728 (3)	0.49053 (2)	0.77186 (2)	0.01071 (6)	
Cl1	0.52632 (12)	0.57653 (3)	0.57191 (7)	0.02214 (17)	
Cl2	0.86113 (15)	0.66386 (3)	1.04825 (9)	0.0310 (2)	
Cl3	0.35058 (15)	0.65895 (3)	1.01756 (8)	0.0320 (2)	
Cl4	0.1852 (2)	0.74928 (3)	0.62045 (10)	0.0402 (3)	
O1	−0.1375 (4)	0.45586 (8)	0.6315 (2)	0.0190 (5)	
H1A	−0.216 (7)	0.4425 (13)	0.666 (4)	0.023*	
H1B	−0.111 (6)	0.4389 (13)	0.586 (4)	0.023*	
O2	0.2230 (4)	0.48617 (8)	0.5597 (2)	0.0201 (5)	
H2A	0.195 (7)	0.4665 (14)	0.522 (4)	0.024*	
H2B	0.227 (6)	0.5050 (14)	0.515 (4)	0.024*	
O3	0.2806 (4)	0.41711 (8)	0.7131 (2)	0.0214 (5)	
H3A	0.234 (7)	0.4074 (13)	0.648 (4)	0.026*	
H3B	0.390 (7)	0.4112 (14)	0.728 (4)	0.026*	
O4	0.5011 (3)	0.51206 (7)	0.7824 (2)	0.0185 (5)	
O5	0.0711 (4)	0.55684 (7)	0.6732 (2)	0.0187 (5)	
O6	0.2443 (4)	0.53789 (8)	0.9496 (2)	0.0162 (5)	
H6A	0.172 (6)	0.5421 (13)	0.994 (4)	0.019*	
H6B	0.365 (7)	0.5414 (13)	0.985 (4)	0.019*	
O7	0.8332 (3)	0.51060 (7)	0.8259 (2)	0.0175 (5)	
O8	0.6424 (3)	0.55065 (7)	1.05147 (19)	0.0160 (5)	
O9	0.9632 (3)	0.56660 (7)	1.08782 (19)	0.0163 (5)	
O10	−0.0218 (4)	0.59396 (8)	0.5099 (2)	0.0203 (5)	
O11	0.1928 (5)	0.66501 (9)	0.4265 (2)	0.0350 (7)	
O12	−0.1099 (4)	0.68329 (9)	0.4598 (2)	0.0321 (6)	
O13	−0.3045 (4)	0.39310 (9)	0.7472 (3)	0.0286 (6)	
H13A	−0.272 (7)	0.3738 (15)	0.711 (4)	0.034*	
H13B	−0.220 (7)	0.3931 (15)	0.814 (4)	0.034*	
C1	0.6686 (5)	0.52900 (10)	0.8040 (3)	0.0139 (6)	
C2	0.6774 (4)	0.57584 (10)	0.8076 (3)	0.0135 (6)	
C3	0.6275 (5)	0.59966 (11)	0.7059 (3)	0.0158 (6)	
C4	0.6544 (5)	0.64206 (11)	0.7079 (3)	0.0208 (7)	
H4	0.622165	0.657699	0.637085	0.025*	
C5	0.7287 (5)	0.66131 (11)	0.8138 (3)	0.0215 (7)	
H5	0.747350	0.690401	0.816744	0.026*	
C6	0.7758 (5)	0.63795 (11)	0.9160 (3)	0.0192 (7)	
C7	0.7527 (5)	0.59551 (10)	0.9149 (3)	0.0138 (6)	
C8	0.7894 (5)	0.56959 (9)	1.0260 (3)	0.0128 (6)	
C9	0.0562 (5)	0.59016 (10)	0.6172 (3)	0.0140 (6)	
C10	0.1326 (5)	0.62886 (10)	0.6833 (3)	0.0153 (6)	
C11	0.1994 (5)	0.62682 (10)	0.8049 (3)	0.0163 (6)	
H11	0.203366	0.601080	0.844882	0.020*	
C12	0.2599 (5)	0.66250 (11)	0.8673 (3)	0.0192 (7)	
C13	0.2529 (6)	0.70049 (11)	0.8124 (3)	0.0235 (7)	
H13	0.290133	0.724962	0.856623	0.028*	
C14	0.1899 (6)	0.70185 (11)	0.6906 (3)	0.0227 (7)	
C15	0.1311 (5)	0.66659 (11)	0.6241 (3)	0.0184 (7)	
C16	0.0752 (6)	0.67031 (11)	0.4919 (3)	0.0235 (7)	
C17	−0.1740 (9)	0.69157 (16)	0.3343 (4)	0.0497 (13)	
H17A	−0.180144	0.665521	0.288787	0.060*	
H17B	−0.078142	0.710400	0.306355	0.060*	
C18	−0.3711 (10)	0.7106 (2)	0.3177 (6)	0.076 (2)	
H18A	−0.418000	0.716488	0.233614	0.114*	
H18B	−0.464813	0.691644	0.345401	0.114*	
H18C	−0.363204	0.736350	0.362901	0.114*	

### Poly[[tetraaqua[2,4-dichloro-6-(ethoxycarbonyl)benzoato](µ3-3,6-dichlorophthalato)lanthanum(III)] monohydrate] Atomic displacement parameters (Å^2^)

**Table:**

	*U*^11^	*U*^22^	*U*^33^	*U*^12^	*U*^13^	*U*^23^
La1	0.00833 (9)	0.01307 (9)	0.01034 (8)	−0.00037 (7)	0.00073 (6)	−0.00107 (7)
Cl1	0.0187 (4)	0.0346 (5)	0.0126 (3)	0.0022 (3)	0.0016 (3)	0.0008 (3)
Cl2	0.0370 (5)	0.0243 (4)	0.0304 (5)	−0.0050 (4)	0.0029 (4)	−0.0104 (4)
Cl3	0.0349 (5)	0.0384 (5)	0.0217 (4)	−0.0004 (4)	0.0030 (4)	−0.0065 (4)
Cl4	0.0657 (8)	0.0160 (4)	0.0377 (5)	−0.0049 (5)	0.0063 (5)	0.0054 (4)
O1	0.0181 (12)	0.0223 (13)	0.0159 (11)	−0.0045 (10)	0.0018 (9)	−0.0044 (10)
O2	0.0268 (13)	0.0183 (13)	0.0158 (12)	−0.0007 (11)	0.0057 (10)	−0.0012 (10)
O3	0.0206 (13)	0.0244 (13)	0.0177 (12)	0.0044 (11)	−0.0003 (10)	−0.0039 (10)
O4	0.0123 (11)	0.0225 (12)	0.0204 (11)	−0.0030 (10)	0.0023 (9)	−0.0013 (10)
O5	0.0223 (12)	0.0144 (11)	0.0197 (11)	0.0004 (10)	0.0046 (10)	0.0012 (9)
O6	0.0098 (11)	0.0244 (13)	0.0144 (11)	0.0002 (10)	0.0024 (9)	−0.0049 (9)
O7	0.0120 (11)	0.0191 (12)	0.0216 (11)	0.0032 (9)	0.0039 (9)	0.0006 (10)
O8	0.0115 (11)	0.0222 (12)	0.0133 (10)	−0.0031 (9)	−0.0001 (8)	0.0017 (9)
O9	0.0108 (10)	0.0227 (12)	0.0148 (11)	0.0010 (9)	0.0009 (9)	0.0008 (9)
O10	0.0220 (12)	0.0208 (12)	0.0163 (11)	0.0026 (10)	−0.0007 (9)	−0.0006 (9)
O11	0.0485 (18)	0.0363 (16)	0.0247 (14)	−0.0026 (14)	0.0189 (13)	0.0013 (12)
O12	0.0415 (17)	0.0302 (15)	0.0211 (13)	0.0112 (13)	−0.0028 (12)	0.0021 (11)
O13	0.0266 (15)	0.0311 (15)	0.0281 (15)	−0.0008 (12)	0.0054 (12)	−0.0013 (12)
C1	0.0136 (15)	0.0200 (16)	0.0089 (13)	−0.0008 (12)	0.0040 (11)	−0.0007 (12)
C2	0.0081 (13)	0.0184 (15)	0.0148 (14)	0.0017 (12)	0.0039 (11)	0.0001 (12)
C3	0.0109 (14)	0.0231 (17)	0.0138 (14)	0.0035 (13)	0.0034 (12)	0.0009 (12)
C4	0.0173 (16)	0.0215 (17)	0.0248 (17)	0.0072 (14)	0.0073 (14)	0.0098 (14)
C5	0.0194 (17)	0.0165 (16)	0.0293 (18)	0.0016 (14)	0.0065 (14)	0.0035 (14)
C6	0.0167 (16)	0.0195 (17)	0.0213 (16)	−0.0018 (13)	0.0034 (13)	−0.0037 (13)
C7	0.0105 (14)	0.0173 (15)	0.0142 (14)	0.0004 (12)	0.0041 (11)	0.0019 (12)
C8	0.0153 (15)	0.0129 (14)	0.0100 (13)	0.0010 (12)	0.0022 (11)	−0.0010 (11)
C9	0.0112 (14)	0.0148 (15)	0.0172 (15)	−0.0007 (12)	0.0058 (12)	−0.0017 (12)
C10	0.0152 (15)	0.0146 (15)	0.0177 (15)	0.0006 (12)	0.0069 (12)	−0.0011 (12)
C11	0.0159 (15)	0.0167 (15)	0.0172 (15)	−0.0009 (13)	0.0058 (12)	0.0012 (12)
C12	0.0174 (16)	0.0247 (18)	0.0151 (15)	0.0001 (14)	0.0024 (12)	−0.0034 (13)
C13	0.0258 (18)	0.0191 (17)	0.0254 (18)	−0.0032 (15)	0.0039 (15)	−0.0057 (14)
C14	0.0292 (19)	0.0148 (16)	0.0252 (18)	0.0003 (14)	0.0078 (15)	0.0024 (14)
C15	0.0190 (16)	0.0192 (16)	0.0175 (16)	0.0013 (13)	0.0050 (13)	0.0019 (13)
C16	0.035 (2)	0.0151 (16)	0.0211 (17)	0.0008 (15)	0.0069 (15)	0.0030 (13)
C17	0.071 (4)	0.046 (3)	0.023 (2)	0.011 (3)	−0.013 (2)	0.0057 (19)
C18	0.065 (4)	0.086 (5)	0.063 (4)	0.016 (4)	−0.022 (3)	0.019 (4)

### Poly[[tetraaqua[2,4-dichloro-6-(ethoxycarbonyl)benzoato](µ3-3,6-dichlorophthalato)lanthanum(III)] monohydrate] Geometric parameters (Å, º)

**Table:**

La1—O4	2.422 (2)	O12—C17	1.454 (5)
La1—O5	2.448 (2)	O13—H13A	0.81 (5)
La1—O7^i^	2.488 (2)	O13—H13B	0.87 (5)
La1—O6	2.537 (2)	C1—C2	1.518 (5)
La1—O2	2.570 (2)	C2—C3	1.390 (4)
La1—O1	2.584 (2)	C2—C7	1.398 (4)
La1—O8^ii^	2.595 (2)	C3—C4	1.385 (5)
La1—O3	2.651 (3)	C4—C5	1.379 (5)
La1—O9^ii^	2.684 (2)	C4—H4	0.9500
La1—C8^ii^	3.004 (3)	C5—C6	1.385 (5)
Cl1—C3	1.737 (3)	C5—H5	0.9500
Cl2—C6	1.740 (4)	C6—C7	1.383 (5)
Cl3—C12	1.729 (3)	C7—C8	1.511 (4)
Cl4—C14	1.733 (4)	C9—C10	1.507 (4)
O1—H1A	0.84 (4)	C10—C11	1.390 (5)
O1—H1B	0.80 (4)	C10—C15	1.398 (5)
O2—H2A	0.77 (5)	C11—C12	1.381 (5)
O2—H2B	0.80 (5)	C11—H11	0.9500
O3—H3A	0.82 (5)	C12—C13	1.379 (5)
O3—H3B	0.76 (5)	C13—C14	1.388 (5)
O4—C1	1.247 (4)	C13—H13	0.9500
O5—C9	1.251 (4)	C14—C15	1.391 (5)
O6—H6A	0.78 (4)	C15—C16	1.504 (5)
O6—H6B	0.85 (4)	C17—C18	1.455 (8)
O7—C1	1.252 (4)	C17—H17A	0.9900
O8—C8	1.255 (4)	C17—H17B	0.9900
O9—C8	1.263 (4)	C18—H18A	0.9800
O10—C9	1.257 (4)	C18—H18B	0.9800
O11—C16	1.210 (5)	C18—H18C	0.9800
O12—C16	1.313 (5)		
			
Cl2^iii^···Cl3	3.4185 (16)	Cl3^iv^···Cl4	3.4645 (15)
Cl2···Cl3	3.4287 (15)		
			
O4—La1—O5	85.14 (8)	O4—C1—O7	125.5 (3)
O4—La1—O7^i^	143.67 (8)	O4—C1—C2	118.4 (3)
O5—La1—O7^i^	75.00 (8)	O7—C1—C2	116.1 (3)
O4—La1—O6	73.11 (8)	C3—C2—C7	118.9 (3)
O5—La1—O6	81.00 (8)	C3—C2—C1	121.9 (3)
O7^i^—La1—O6	73.93 (8)	C7—C2—C1	119.0 (3)
O4—La1—O2	73.96 (8)	C4—C3—C2	121.7 (3)
O5—La1—O2	71.11 (8)	C4—C3—Cl1	118.1 (3)
O7^i^—La1—O2	125.01 (8)	C2—C3—Cl1	120.2 (3)
O6—La1—O2	138.13 (8)	C5—C4—C3	119.2 (3)
O4—La1—O1	141.71 (8)	C5—C4—H4	120.4
O5—La1—O1	90.05 (8)	C3—C4—H4	120.4
O7^i^—La1—O1	69.51 (8)	C4—C5—C6	119.5 (3)
O6—La1—O1	143.43 (8)	C4—C5—H5	120.2
O2—La1—O1	68.58 (8)	C6—C5—H5	120.2
O4—La1—O8^ii^	75.46 (7)	C7—C6—C5	121.8 (3)
O5—La1—O8^ii^	149.22 (8)	C7—C6—Cl2	120.4 (3)
O7^i^—La1—O8^ii^	107.29 (7)	C5—C6—Cl2	117.8 (3)
O6—La1—O8^ii^	70.69 (8)	C6—C7—C2	118.9 (3)
O2—La1—O8^ii^	123.90 (8)	C6—C7—C8	122.9 (3)
O1—La1—O8^ii^	119.92 (8)	C2—C7—C8	118.0 (3)
O4—La1—O3	85.50 (8)	O8—C8—O9	122.2 (3)
O5—La1—O3	136.70 (8)	O8—C8—C7	117.2 (3)
O7^i^—La1—O3	129.51 (8)	O9—C8—C7	120.5 (3)
O6—La1—O3	135.31 (8)	O8—C8—La1^ii^	59.15 (16)
O2—La1—O3	65.67 (8)	O9—C8—La1^ii^	63.26 (16)
O1—La1—O3	72.34 (8)	C7—C8—La1^ii^	173.1 (2)
O8^ii^—La1—O3	66.07 (8)	O5—C9—O10	124.8 (3)
O4—La1—O9^ii^	124.74 (7)	O5—C9—C10	118.0 (3)
O5—La1—O9^ii^	144.12 (7)	O10—C9—C10	117.2 (3)
O7^i^—La1—O9^ii^	69.12 (7)	C11—C10—C15	120.2 (3)
O6—La1—O9^ii^	88.95 (7)	C11—C10—C9	119.1 (3)
O2—La1—O9^ii^	131.49 (8)	C15—C10—C9	120.7 (3)
O1—La1—O9^ii^	77.78 (7)	C12—C11—C10	119.5 (3)
O8^ii^—La1—O9^ii^	49.35 (7)	C12—C11—H11	120.2
O3—La1—O9^ii^	71.52 (7)	C10—C11—H11	120.2
O4—La1—C8^ii^	99.99 (8)	C13—C12—C11	121.8 (3)
O5—La1—C8^ii^	155.69 (8)	C13—C12—Cl3	119.5 (3)
O7^i^—La1—C8^ii^	87.61 (8)	C11—C12—Cl3	118.7 (3)
O6—La1—C8^ii^	77.89 (8)	C12—C13—C14	117.9 (3)
O2—La1—C8^ii^	133.19 (8)	C12—C13—H13	121.0
O1—La1—C8^ii^	99.77 (8)	C14—C13—H13	121.0
O8^ii^—La1—C8^ii^	24.53 (8)	C13—C14—C15	122.2 (3)
O3—La1—C8^ii^	67.60 (8)	C13—C14—Cl4	118.3 (3)
O9^ii^—La1—C8^ii^	24.86 (8)	C15—C14—Cl4	119.5 (3)
La1—O1—H1A	114 (3)	C14—C15—C10	118.3 (3)
La1—O1—H1B	118 (3)	C14—C15—C16	118.9 (3)
H1A—O1—H1B	101 (4)	C10—C15—C16	122.7 (3)
La1—O2—H2A	120 (3)	O11—C16—O12	125.6 (4)
La1—O2—H2B	127 (3)	O11—C16—C15	123.2 (4)
H2A—O2—H2B	108 (4)	O12—C16—C15	110.9 (3)
La1—O3—H3A	120 (3)	O12—C17—C18	107.8 (5)
La1—O3—H3B	121 (3)	O12—C17—H17A	110.2
H3A—O3—H3B	107 (4)	C18—C17—H17A	110.2
C1—O4—La1	167.0 (2)	O12—C17—H17B	110.2
C9—O5—La1	169.9 (2)	C18—C17—H17B	110.2
La1—O6—H6A	123 (3)	H17A—C17—H17B	108.5
La1—O6—H6B	122 (3)	C17—C18—H18A	109.5
H6A—O6—H6B	109 (4)	C17—C18—H18B	109.5
C1—O7—La1^iii^	151.1 (2)	H18A—C18—H18B	109.5
C8—O8—La1^ii^	96.32 (18)	C17—C18—H18C	109.5
C8—O9—La1^ii^	91.88 (18)	H18A—C18—H18C	109.5
C16—O12—C17	115.5 (3)	H18B—C18—H18C	109.5
H13A—O13—H13B	105 (5)		
			
La1—O4—C1—O7	133.7 (9)	C6—C7—C8—O9	−65.7 (4)
La1—O4—C1—C2	−44.7 (11)	C2—C7—C8—O9	119.3 (3)
La1^iii^—O7—C1—O4	111.9 (4)	La1—O5—C9—O10	90.4 (13)
La1^iii^—O7—C1—C2	−69.7 (5)	La1—O5—C9—C10	−91.1 (13)
O4—C1—C2—C3	−70.8 (4)	O5—C9—C10—C11	−5.8 (4)
O7—C1—C2—C3	110.6 (3)	O10—C9—C10—C11	172.9 (3)
O4—C1—C2—C7	114.2 (3)	O5—C9—C10—C15	176.1 (3)
O7—C1—C2—C7	−64.3 (4)	O10—C9—C10—C15	−5.2 (4)
C7—C2—C3—C4	1.4 (5)	C15—C10—C11—C12	1.5 (5)
C1—C2—C3—C4	−173.6 (3)	C9—C10—C11—C12	−176.6 (3)
C7—C2—C3—Cl1	−178.0 (2)	C10—C11—C12—C13	1.1 (5)
C1—C2—C3—Cl1	7.0 (4)	C10—C11—C12—Cl3	−177.7 (2)
C2—C3—C4—C5	−1.4 (5)	C11—C12—C13—C14	−2.4 (5)
Cl1—C3—C4—C5	178.0 (3)	Cl3—C12—C13—C14	176.4 (3)
C3—C4—C5—C6	0.3 (5)	C12—C13—C14—C15	1.2 (6)
C4—C5—C6—C7	0.8 (5)	C12—C13—C14—Cl4	−178.8 (3)
C4—C5—C6—Cl2	−177.9 (3)	C13—C14—C15—C10	1.2 (5)
C5—C6—C7—C2	−0.8 (5)	Cl4—C14—C15—C10	−178.7 (3)
Cl2—C6—C7—C2	177.9 (2)	C13—C14—C15—C16	−176.8 (3)
C5—C6—C7—C8	−175.7 (3)	Cl4—C14—C15—C16	3.2 (5)
Cl2—C6—C7—C8	3.0 (4)	C11—C10—C15—C14	−2.6 (5)
C3—C2—C7—C6	−0.3 (4)	C9—C10—C15—C14	175.5 (3)
C1—C2—C7—C6	174.8 (3)	C11—C10—C15—C16	175.4 (3)
C3—C2—C7—C8	174.9 (3)	C9—C10—C15—C16	−6.5 (5)
C1—C2—C7—C8	−10.0 (4)	C17—O12—C16—O11	0.2 (6)
La1^ii^—O8—C8—O9	−4.8 (3)	C17—O12—C16—C15	174.7 (3)
La1^ii^—O8—C8—C7	173.3 (2)	C14—C15—C16—O11	93.0 (5)
La1^ii^—O9—C8—O8	4.7 (3)	C10—C15—C16—O11	−85.0 (5)
La1^ii^—O9—C8—C7	−173.4 (2)	C14—C15—C16—O12	−81.6 (4)
C6—C7—C8—O8	116.1 (3)	C10—C15—C16—O12	100.4 (4)
C2—C7—C8—O8	−58.8 (4)	C16—O12—C17—C18	−172.7 (4)

Symmetry codes: (i) *x*−1, *y*, *z*; (ii) −*x*+1, −*y*+1, −*z*+2; (iii) *x*+1, *y*, *z*; (iv) *x*, −*y*+3/2, *z*+1/2.

### Poly[[tetraaqua[2,4-dichloro-6-(ethoxycarbonyl)benzoato](µ3-3,6-dichlorophthalato)lanthanum(III)] monohydrate] Hydrogen-bond geometry (Å, º)

**Table:**

*D*—H···*A*	*D*—H	H···*A*	*D*···*A*	*D*—H···*A*
O1—H1*A*···O13	0.84 (4)	2.00 (4)	2.787 (4)	155 (4)
O1—H1*B*···O10^v^	0.80 (4)	1.89 (5)	2.663 (4)	164 (4)
O2—H2*A*···O10^v^	0.77 (5)	2.28 (5)	2.972 (4)	150 (4)
O2—H2*B*···O1^v^	0.80 (5)	2.11 (5)	2.868 (4)	159 (4)
O3—H3*A*···O10^v^	0.82 (5)	2.10 (5)	2.850 (4)	152 (4)
O3—H3*B*···O13^iii^	0.76 (5)	2.13 (5)	2.883 (4)	174 (5)
O6—H6*A*···O9^i^	0.78 (4)	2.10 (4)	2.864 (3)	166 (4)
O6—H6*B*···O8	0.85 (4)	1.92 (4)	2.772 (3)	175 (4)
O13—H13*A*···O11^v^	0.81 (5)	2.16 (5)	2.948 (4)	164 (5)
O13—H13*B*···O9^ii^	0.87 (5)	2.30 (5)	3.008 (4)	139 (4)

Symmetry codes: (i) *x*−1, *y*, *z*; (ii) −*x*+1, −*y*+1, −*z*+2; (iii) *x*+1, *y*, *z*; (v) −*x*, −*y*+1, −*z*+1.

### Continuous Shape Measurements (CShM) for [La(3,6-dcpa)(C_10_H_7_Cl_2_O_4_)(H_2_O)_4_.H_2_O]_∞_. The lower is the CShM value, the better is the agreement with the given coordination polyhedron.

**Table:**

[ML9]	EP-9	OPY-9	HBPY-9	JTC-9	JCCU-9	CCU-9	JCSAPR-9	CSAPR-9	JTCTPR-9	TCTPR-9	JTDIC-9	HH-9	MFF-9
La	35.863	21.416	20.115	15.640	11.075	9.311	2.176	1.059	2.715	1.073	13.106	10.786	1.588

EP-9 ≡*D*_9_*_h_*-Enneagon; OPY-9 ≡*C*_8_*_v_*-Octagonal pyramid; HBPY-9 ≡*D*_7_*_h_*-Heptagonal bipyramid; JTC-9≡*C*_3_*_v_*-Johnson triangular cupola J3; JCCU-9 ≡*C*_4_*_v_*-Capped cube J8; CCU-9 ≡*C*_4_*_v_*-Spherical-relaxed capped cube; JCSAPR-9 *C*_4_*_v_*-Capped square antiprism J10; CSAPR-9 ≡*C*_4_*_v_*-Spherical capped square antiprism; JTCTPR-9 ≡*D*_3_*_h_*-Tricapped trigonal prism J51; TCTPR-9 ≡*D*_3_*_h_* Spherical tricapped trigonal prism; JTDIC-9 ≡*C*_3_*_v_*-Tridiminished icosahedron J63; HH-9 ≡*C*_2_*_v_*-Hula-hoop; MFF-9 ≡*C_s_*-Muffin.
